# Novel Standard Substance from Primary Hamster Kidney Cells for Quality Control of Human Rabies Vaccines in China

**DOI:** 10.3390/vaccines13020180

**Published:** 2025-02-13

**Authors:** Leitai Shi, Jia Li, Xiaohong Wu, Shouchun Cao, Yunpeng Wang, Danhua Zhao, Yuhua Li

**Affiliations:** National Institutes for Food and Drug Control, Beijing 102629, China; taige@nifdc.org.cn (L.S.); lijiarv@nifdc.org.cn (J.L.); wuxiaohong@nifdc.org.cn (X.W.); caosc@nifdc.org.cn (S.C.); wangyunpeng@nifdc.org.cn (Y.W.); zhaodanhua@nifdc.org.cn (D.Z.)

**Keywords:** host cell protein, primary hamster kidney cell, protein standard, human rabies vaccine, quality control

## Abstract

Background: Host cell proteins (HCPs) from primary hamster kidney cells (PHKCs) used to produce rabies vaccines may cause an allergic reaction in humans, so these residual HCPs must be controlled. Establishing a national standard for PHKC HCP is very important to ensure the consistency of HCPs between batches of the vaccine and to standardize the control of HCPs. Objectives: We aimed to establish a novel national standard substance to determine the HCP residue in rabies vaccines produced with PHKCs. Methods: A two-step multi-laboratory collaborative collaboration was undertaken. In the first step, the protein concentration of the standard substance stock solution was determined using Lowry’s method. In the second step, the concentration of the candidate standard substance was determined with an enzyme-linked immunosorbent assay. Results: The concentration of the PHKC protein standard was 4.0 μg/mL (95% confidence interval: 3.5–4.4 μg/mL). Conclusions: The PHKC protein standard was approved by the Chinese National Committee on Standards for the quality control of PHKC-based rabies vaccines for human use, and it plays an important role in controlling the quality of these human vaccines.

## 1. Introduction

Rabies is a kind of traditional zoonotic disease widely distributed in over 150 countries and regions. Globally, approximately 29 million people need to receive post-exposure prophylaxis and vaccination against rabies each year, resulting in an estimated economic loss of 8.6 billion USD annually. Rabies is preventable and controllable, and vaccine inoculation is the most efficacious and economic strategy for humans to prevent rabies [1]. According to the Notice on Issuing the Work Norms for Rabies Exposure Prevention and Treatment (2023 Edition), individuals who need to be vaccinated can attend rabies prevention and treatment clinics set up within the jurisdiction of health and disease control departments at or above the county level for vaccination in China.

The first type of rabies vaccine invented by Pasteur in 1885 was made of nerve tissue, which was used for humans for over 70 years [2]. Adverse reactions to the vaccine with neural tissue residue were often reported. The solution to the problem was to seek a type of rabies vaccine free from neural tissue. The first attempts to manufacture a rabies vaccine in non-neural tissue were made by Kissling in 1958 [3,4] and by Fenje in 1960 [5]. Both investigators used primary hamster kidney (PHK) cells for rabies virus preparation. Over the past few decades, a variety of substrates, such as whole animal tissues, primary cell cultures, diploid cells, and continuous cell lines, have been used widely for rabies virus propagation and rabies vaccine production for human use. Interestingly, all of these systems remain in use today for human rabies vaccine production [2].

In 1980, Chinese scientists successfully developed a primary hamster kidney cell-based vaccine (PHKCV) [6,7,8] with the RABV Beijing (aG) strain [9,10]. This rabies PHKCV is still available and still used in China and plays an active role in protecting humans from rabies. The main component of this PHKCV for human use is an RABV antigen of strain aG combined with a stabilizer. However, it is inevitable that components of the host cell proteins (HCPs) remain in the vaccine, and these residual proteins may cause an allergic reaction in humans, so these residual HCPs must be monitored.

With the development and progress of biologic technology, the host cell residues could be removed to enhance the purity of the vaccine and to monitor the consistency of the production processes [11]. These residues include host cell DNA and host cell endogenous proteins, known as host cell proteins (HCPs) [12]. HCPs could affect product quality, efficacy, and safety [13]. Anti-HCP enzyme-linked immunosorbent assay (HCP ELISA) has often been the standard method for measuring HCPs, which can quickly evaluate the level of HCP residues [14]. HCP ELISA provides a simple immunological measurement method for total impurity levels [11].

At present, ELISA is used to determine the levels of HCP in PHKCVs in China, and the limit is ≤24 μg/dose [15]. However, the standard substance for an HCP residue test was not available in China, or even in the world. Nowadays, the standard substance used in this test is contained in the ELISA kit. To standardize the test on HCPs and ensure lot-to-lot consistency of HCPs in PHKCVs, it is very important to establish a national standard substance for the PHKC HCP test.

## 2. Materials and Methods

### 2.1. Experimental Materials

The primary hamster kidney cells (specific-pathogen-free, SPF) were obtained from the Beijing Institute of Biological Products Co., Ltd. (BIBP) (Beijing, China). The experimental data in the manuscript were not derived from experimental animals. The primary hamsters were only used as raw materials for standard substance which complied with related requirements in China. The trypsin-EDTA was purchased from the Life Technologies Corporation (Grand Island, NE, USA). The minimum essential medium (MEM) was purchased from the Life Technologies Corporation (Grand Island, NE, USA). The penicillin–streptomycin liquid and phosphate buffered saline (PBS) were purchased from Beijing Solarbio Science & Technology Co., Ltd. (Beijing, China). The fetal bovine serum was purchased from Thermo Fisher Scientific New Zealand Limited (Auckland, New Zealand).

The national standard substance for determining the HCP content in PHKCVs (code numbers: 270009–201107; 19.73 mg/amp) was purchased from the National Control Laboratory (NCL) to quantify the protein in the stock solution. The national standard substance was diluted with distilled water to 200 µg/mL. The alkaline copper solution was sourced from the National Institutes for Food and Drug Control (NIFDC) (Beijing, China). The Folin–Ciocalteu’s phenol reagent was purchased from Sigma-Aldrich (Shanghai, China) Trading Co., Ltd. (Shanghai, China). The UV–Vis spectrophotometer was purchased from SHIMADZU (China) Co., Ltd. (Suzhou, China).

The validated ELISA kit was prepared by the BIBP (Beijing, China). Lots 20190412 and 20190413 were used for the collaborative calibration of the candidate standard by four laboratories. Lots 20190414 and 20190415 were used to verify the applicability of the standard substance for the measurement of HCPs in PHKCVs. Six batches of applicability validation samples were obtained from two domestic manufacturers of rabies vaccines prepared with primary hamster kidney cells. The microplate reader was purchased from Tecan Austria GmbH (Grödlg, Austria).

### 2.2. Raw Material and Stock Solution

The raw material used to produce the candidate standard substance was obtained from the BIBP (Beijing, China) and was prepared according to the principles of Good Manufacturing Practice and the guidelines of the Pharmacopoeia of the People’s Republic of China. Briefly, the kidneys of SPF hamsters were dissected aseptically, the kidney tissue was digested with trypsin, and the cells were prepared and cultured with MEM. After the cells grew into a dense monolayer at 34–35 °C, the medium was changed, and the cells continued to be cultured for 4 days. The culture supernatant was harvested. The secreted proteins in the supernatant were concentrated with 100 kD ultrafiltration and collected. This protein solution was the raw material. The stock solution was prepared by diluting it fivefold.

### 2.3. Candidate Standard Substance

**Figure 1:**
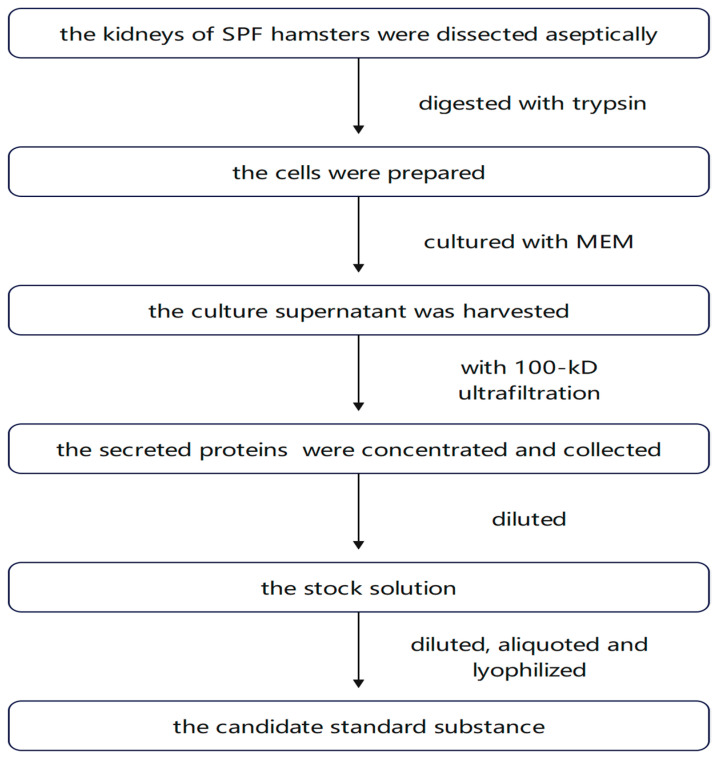
Process steps of the candidate standard substance.

The stock solution was diluted, aliquoted, and lyophilized. The lyophilized product was prepared in ampoules and reconstituted with 0.5 mL of sterile water for injections as required. The process steps are shown in Figure 1.

### 2.4. Collaborative Calibration of the Candidate Standard Substance

The NIFDC designed and distributed the standard operating procedure to the laboratories for the collaborative calibration of the candidate standard substance. All of the laboratories used the same procedure.

In the first step, five qualified laboratories determined the protein content of the stock solution with the Lowry assay. The protein standard solution was accurately measured as 0.0 mL, 0.2 mL, 0.4 mL, 0.6 mL, 0.8 mL, and 1.0 mL into stoppered test tubes. Distilled water was added to each tube to 1.0 mL, and then 1.0 mL of alkaline copper solution was added. The tubes were shaken well and allowed to stand at room temperature for 10 min. A total of 4.0 mL of Folin–Ciocalteu’s phenol reagent was added per tube and mixed immediately. The tubes were left at room temperature for 30 min. The same method was used to accurately measure the appropriate amount of the stock solution. The absorbance was read at 650 nm using a UV–Vis spectrophotometer. The dilution of the standard was taken as the horizontal axis, and the absorbance was taken as the vertical axis; the standard curve was plotted. The linear regression equation method was used to fit the curve and calculate the protein content of the stock solution. Each laboratory reported data from three independent analyses, so a total of 15 datasets were collected by the NIFDC, and a value was assigned to the stock solution after a statistical analysis.

In the second step, the stock solution was serially diluted to different concentration points (200, 100, 50, 25, 12.5, and 6.25 ng/mL), which were used to construct a standard curve for the ELISA, with which we calculated the HCP concentration. A sandwich ELISA was used to detect the concentration of the HCPs in the samples. The standard and sample from each process stage were diluted serially and added to the reaction plate. After incubation, they were combined with enzyme-labeled antibodies against HCPs, followed by the addition of a chromogenic substrate solution and a stop solution. The absorbance was read at a wavelength of 450 nm using a microplate reader. A standard curve was plotted with the dilution of the standard on the horizontal axis and the absorbance of the standard on the vertical axis. The curve was fitted using a linear regression equation method to calculate the concentration of HCPs in the sample. Four laboratories were invited to participate in the collaborative calibration, and each laboratory reported 10 sets of data (2 kits from different lots, 5 analyses per kit), so a total of 40 datasets were collected by the NIFDC.

### 2.5. Verification of the Applicability of the Candidate Standard

The PHKC HCP standard substance was used to determine the HCP residue in rabies PHKCVs for human use with an ELISA. The candidate standard was diluted to six concentration points (200, 100, 50, 25, 12.5, and 6.25 ng/mL) to plot the ELISA standard curve. Please refer to Section 2.4 HCP ELISA for the detailed procedure.

In the applicability verification process, two vaccine manufacturers each provided PHKCVs from three different lots. The six lots of the vaccine were coded blindly and distributed to four laboratories. Their release was authorized by the NCL. Each laboratory reported 6 sets of data (2 kits from different lots, 3 analyses per kit), so a total of 24 datasets were collected.

### 2.6. Inspection of the Stability of the HCP Standard Derived from PHKCs

Both the short- and long-term stability of the standard substances are tested. A long-term stability test monitors and accumulates data over a long period during the use of the product, whereas “short-term stability” refers to the stability of a standard substance under transportation conditions. The bioactivity assay was performed at different temperatures (4 °C, 25 °C, and 37 °C) and different times (1, 2, 3, and 4 weeks) to evaluate the stability. The biological activity of the HCP standards was determined using an ELISA. The HCP standards were diluted to different concentration points (200, 100, 50, 25, 12.5, and 6.25 ng/mL), and the ELISA standard curve was plotted. Please refer to Section 2.4 HCP ELISA for the detailed procedure.

In this study, we only examined the short-term stability of the standard substance. A variance analysis is the method most commonly used to measure stability. The method compares the within-group and between-group variance. If the within-group variance/between-group variance ratio is less than the critical value, the sample is considered to be stable [16,17].

### 2.7. Samples for the Collaborative Calibration Study

The samples used in this study were prepared and distributed by the NIFDC. Information on the samples is given in Table 1.

### 2.8. Data Summary and Analysis of Results

The NIFDC collected and analyzed the protein content data from the collaborative calibration of the stock solution and the standard substance and from the verification of its applicability.

## 3. Results

### 3.1. Protein Content Determination of the Stock Solution of the Candidate Standard Substance

The protein content of the stock solution of the candidate standard substance derived from PHKCs was determined using the Lowry assay. The coefficients of variation for the determinations made by five laboratories ranged from 0% to 9%, all of which were <10%, so the results were acceptable. The candidate standard substance was assigned a protein concentration of 15 μg/mL (Table 2).

### 3.2. Protein Content of the PHKC-Derived Standard Substance

The protein content of the PHKC standard substance was determined with ELISA using the kit described in Section 2.1. All data were analyzed statistically with the Statistical Package for the Social Sciences (SPSS, version 25.0; IBM Corp., Armonk, NY, USA) after outliers were removed. The protein content of the PHKC standard substance was assigned a value of 4.0 μg/mL by four laboratories using ELISA kits from two different lots (Table 3), with a 95% confidence interval (CI) of 3.5–4.4 μg/mL.

### 3.3. Applicability Verification of the PHKC Protein Standard

The PHKC protein standard was serially diluted from 200 to 6.25 ng/mL. Two ELISA kits from different lots and six lots of PHKCVs from two vaccine manufacturers were randomly selected to verify the applicability of the HCP standard for use in these tests (Table 4).

The HCP contents shown in Table 4 were consistent with those determined by the manufacturers themselves (the results are not presented in this text) and by the NCL (using the standard contained in ELISA kits from the same lots to determine the HCP content in the same six lots of the vaccine).

### 3.4. Stability of PHKC Protein Standard

The candidate standard substance was placed at different temperatures (4, 25, or 37 °C) for different periods of time (1, 2, 3, and 4 weeks). Three ampoules were removed at different time points and stored at −20 °C. The biological activity of the substance was determined with an ELISA to evaluate its stability (Table 5).

A variance analysis was used to evaluate the stability of the standard substance [16,17]. According to the degrees of freedom and the given significance level (α = 0.05), the critical value of analysis of variance (ANOVA) was F_0.05_ = 4.07 in the F table. A statistical analysis showed that F1 = 0.78 when the stability was tested at 4 °C, F2 = 1.10 when tested at 25 °C, and F3 = 0.90 when tested at 37 °C. The comparison showed that F1 < F_0.05_, F2 < F_0.05_, and F3 < F_0.05_ (Table 6), so there was no significant difference between the within-group and between-group variance, and the standard substance remained stable at various temperatures (4, 25, and 37 °C) and for different periods (1, 2, 3, and 4 weeks).

## 4. Discussion

The number of human deaths globally due to dog-transmitted rabies is estimated to be about 59,000 annually [18]. Although the status of the rabies epidemic in China has improved in recent years, the situation is not good, and rabies still poses a threat to human health. Primary hamster kidney cell (PHKC)-derived human rabies vaccines (PHKCVs) have been used to prevent rabies in China for half a century [6,7,8], and this kind of vaccine is still available on the Chinese market. Between 2018 and 2020, approximately 5.34 million, 3.61 million, and 5.27 million doses of PHKCV were released annually for human use in China [19], respectively. Therefore, the effectiveness and safety of PHKCVs are particularly important. The results showed that rabies vaccines produced with the aG strain of primary hamster kidney cells rapidly produced protective antibodies after immunization in humans. A retrospective analysis showed that all rabies vaccines were compliant and had good immunogenicity [20]. To ensure their safety, it is necessary to control the residues derived from the host cells of the vaccine virus.

The results showed that rabies vaccines produced with the aG strain of primary hamster kidney cells rapidly produced protective antibodies after immunization in humans. A retrospective analysis showed that all rabies vaccines were compliant and had good immunogenicity.

When present as an impurity in a vaccine, HCPs may induce an allergic reaction in the vaccinated subject, which may trigger unnecessary immune responses that affect the efficacy of the vaccine or even cause hypersensitivity or other adverse reactions. Therefore, it is necessary to control the HCP residues in PHKCVs. ELISA is an important assay for the high-throughput monitoring of HCPs, with high sensitivity and a limit of detection of about 1 ppm. It is relatively easy to perform in standard analytical laboratories.

Although there is no clear definition of HCPs in the World Health Organization (WHO) guidelines [21,22], China’s drug regulatory authorities are especially mindful of the safety of the vaccines. China was the first country in the world to insist that the HCP residues in vaccines for human use should be controlled, and the acceptable limit for HCP residues derived from PHKCs in human rabies vaccines was defined as ≤24 μg/dose [15], which was effectively measured with a validated ELISA [23,24], as reported in the Pharmacopoeia of the People’s Republic of China (Volume III, 2010 Edition). This limit of acceptable HCP impurities and its determination are important in the technical support and supervision required to control the quality of human rabies PHKCVs. However, there is no national standard at present in China, or even the world, for this purpose, and only the standard substance provided with the ELISA kit can be used to assay HCP contamination. Therefore, the establishment of a national standard for PHKC HCPs is urgently required.

To ensure the continuity of quality control, we used two different lots of ELISA kits for the collaborative calibration of the standard substance with an ELISA. A standard curve (6.25–200 ng/mL) was constructed for the PHKC stock solution, and four laboratories were invited to participate in the collaborative calibration. Each participating laboratory tested the sample independently. Each laboratory reported at least ten datasets (with kits from two different lots, five repetitions per kit). The 38 effective datasets collected were analyzed statistically, and the standard concentration of the PHKC HCP standard was determined to be 4.0 μg/mL (95% CI: 3.5–4.4 μg/mL).

The PHKC HCP standard was used to verify the applicability of six different lots of human rabies PHKCVs from two manufacturers in China. The verification results showed that the manufacturers of PHKCVs for human use met the new regulatory requirements and that the HCP residues also met the national quality standard, so no change to the production process was necessary. Thus, a smooth transition to the national standard, replacing the standard substance contained in the kit, was realized.

The stability of the PHKC protein standard was evaluated with a variance analysis. The standard substance showed satisfactory stability.

The PHKC protein standard has several advantages over the standard contained in the ELISA kit. The kit standard fluctuates between lots, whereas the national PHKC protein standard is consistently prepared and shows good uniformity and stability. These advantages ensure that the determination of HCPs will remain unchanged for a considerable period of time, which will allow tests to be standardized and the quality of rabies PHKCVs to be controlled. Furthermore, the establishment and use of the novel PHKC protein standard will allow a smooth transition from the old to the new standard. The first lots of the national PHKC protein standard substance (lot numbers 250024–201901) for the determination of the HCP residues in human rabies PHKCVs have successfully been produced and assigned a concentration of 4.0 μg/mL, with good application verification.

## 5. Conclusions

In this study, the primary hamster kidney cell protein standard (lot No. 250024-201901) was successfully established, which has been assigned to 4.0 μg/mL, completed the applicability verification, and also passed the stability investigation. It has been used for quality control and product release of human rabies vaccine residual protein since 2019 and plays an important role in standardizing the quality control of human vaccines based on primary hamster kidney cells. Currently and in the future, the primary hamster kidney cell protein standard will play an important role in the quality control of human rabies vaccines. It also has important guiding significance for the standardization of residual protein control of human vaccines.

## Figures and Tables

**Table 1:** Information on the samples subjected to collaborative calibration by several laboratories.

Lab No.	Sample	Purpose	Type	Content	Number/Lab
1	National standard substance for protein content determination (270009-201107)	Stock solution assignment	Lyophilized	19.73 mg/amp	1
2	Primary hamster kidney protein stock solution to be calibrated	Stock solution calibration	Liquid	to be calibrated	13
3	Primary hamster kidney protein standard to be calibrated	Standard calibration	Lyophilized	to be calibrated	10
4	Applicability verification sample H1	Standard verification	Liquid	to be verified	6
5	Applicability verification sample H2	Standard verification	Liquid	to be verified	6
6	Applicability verification sample H3	Standard verification	Liquid	to be verified	6
7	Applicability verification sample I1	Standard verification	Liquid	to be verified	6
8	Applicability verification sample I2	Standard verification	Liquid	to be verified	6
9	Applicability verification sample I3	Standard verification	Liquid	to be verified	6

**Table 2:** Collaborative results for the protein content of the PHKC-derived standard.

Lab No.	Contents (μg/mL)			Average Contents (μg/mL)	SD	CV(%)
	1	2	3			
1	15	16	15	15	1	7
2	19	19	18	19	1	5
3	11	12	10	11	1	9
4	18	18	18	18	0	0
5	13	13	11	12	1	8
Assignment protein content of PHKC standard substance stock solution				15	/	/

**Table 3:** Protein contents of PHKC-derived standard substance determined collaboratively by several laboratories.

Lab No.	KIT Lot No.	Determination Times					Average (μg/mL)
		1	2	3	4	5	
A	20190412	2.8	3.0	2.4	2.5	3.1	2.8
	20190413	3.1	3.1	2.8	2.7	3.0	2.9
B	20190412	4.6	4.6	6.4	2.2	3.2	4.2
	20190413	7.4 *	4.5	5.0	5.1	4.7	4.8
C	20190412	4.1	4.1	3.1	3.8	3.3	3.7
	20190413	4.4 *	3.5	3.6	3.8	3.2	3.5
D	20190412	6.4	6.3	6.3	2.9	3.1	5.0
	20190413	6.6	6.6	6.6	2.8	3.0	5.1
Assignment protein content of PHKC standard substance							4.0

* Data were abnormal and not included in the analysis.

**Table 4:** Applicability verification of the PHKC protein standard.

Lab No.	Sample No.	Kit Lot No.							
		20190414				20190415			
		Determination Times			Average Content/(μg/dose)	Determination Times			Average Content/(μg/dose)
		1	2	3		1	2	3	
A	H1	11	11	10	11	10	9	11	10
	H2	15	15	15	15	14	13	16	14
	H3	10	11	10	10	10	9	11	10
	I1	22	22	22	22	20	20	22	21
	I2	21	21	20	21	20	19	21	20
	I3	22	21	21	21	20	19	22	20
B	H1	11	12	11	11	13	12	11	12
	H2	13	17	14	15	18	13	13	15
	H3	9	12	9	10	14	12	10	12
	I1	22	21	21	21	23	22	21	22
	I2	20	22	22	21	22	23	22	22
	I3	19	21	21	20	22	21	20	21
C	H1	8	8	8	8	8	8	9	8
	H2	12	11	14	12	10	11	12	11
	H3	8	8	8	8	8	8	8	8
	I1	15	16	17	16	15	16	17	16
	I2	15	16	15	15	14	14	17	15
	I3	14	16	14	15	15	15	15	15
D	H1	8	7	8	8	8	7	7	7
	H2	10	10	12	11	10	10	10	10
	H3	7	7	7	7	7	7	7	7
	I1	15	16	18	16	17	16	16	16
	I2	16	15	16	16	16	16	16	16
	I3	14	14	17	15	16	15	15	15

**Table 5:** Stability of PHKC protein standard.

Points of Time	Determined Content at Different Temperatures (μg/mL)								
	4 °C			25 °C			37 °C		
1 week	4.0	3.8	3.9	3.9	3.9	3.9	4.0	4.0	4.0
2 weeks	4.0	4.0	4.1	3.9	4.1	4.0	4.0	4.0	4.1
3 weeks	4.0	3.8	4.1	3.8	4.1	3.7	4.1	4.0	4.1
4 weeks	4.0	3.8	4.0	3.8	3.9	3.8	4.1	4.0	4.0

**Table 6:** Variance analysis of the stability of PHKC protein standard.

Statistical Parameters	Statistic		
	4 °C	25 °C	37 °C
Mean	3.96	3.90	4.03
Sum of variance intra-group Q1	0.0292	0.046666667	0.0068
Sum of variance inter-group Q2	0.1001	0.1134	0.0201
S1	0.00973	0.01556	0.00227
S2	0.01251	0.01418	0.00251
F	0.78	1.10	0.90
Critical F_0.05_	4.07	4.07	4.07
Conclusion	F < F_0.05_, there was no significant difference between the within-group and between-group variance.		

## Data Availability

The data presented in this study are available on request from the corresponding author.
